# Supplementary material for: Towards a better understanding of the low recall of insertion variants with short-read based variant callers
Source: BMC Genomics. 2020 Nov 4;21:762. doi: 10.1186/s12864-020-07125-5 (PMC7640490; doi:10.1186/s12864-020-07125-5)
Supplement: Supplementary file 1 — Additional file 1 Supplementary Figures and Tables. [file 12864_2020_7125_MOESM1_ESM.pdf]

ADDITIONAL FILE

Additional file for: Towards a better understanding of the low recall of insertion variants with short-read based variant callers

Wesley J Delage[1*], Julien Thevenon[2] and Claire Lemaitre[1]

*Correspondence: wesley.delage@irisa.fr
[1]Univ Rennes, CNRS, Inria, IRISA, UMR 6074, F-35000 Rennes, France
Full list of author information is available at the end of the article

Contents

1 Characteristics of the studied callsets **2**

2 Characterization of insertion callsets **3**
2.1 Annotation of insertions . . . 3
2.2 Distributions of insertion variant features across several callsets . . . 3
2.3 Proportions of SR-based insertion discoveries according to insertion features . . . 4

3 Additional simulation results **5**
3.1 Recall of SV callers without any quality filter . . . 5
3.2 False positive amounts . . . 6
3.3 Insertion recall of short read vs long read SV callers . . . 7

1 Characteristics of the studied callsets

Table 1 Sequencing technologies, sequencing coverage and SV callers used to generate the four high confidence SV callsets that were studied in this work.

Study	Individual	Sequencing technology	Sequence coverage	SV discovery	SV validation
Chaisson et al 2019 [1]	NA19240<br>HG00514<br>HG00733	Illumina short insert<br>Illumina liWGS<br>Illumina 7kbp JMP	77<br>3<br>1	dCGH, Delly, GenomeStrip, NovoBreak,Pindel, retroCNV, SVelter, VH, Wham, Lumpy, ForestSV, Manta, MELT, Tardis_MEI, liWGS	Long read alignment<br>Optical mapping
		10X Chromium<br>BioNanoGenomics<br>Tru-Seq SLR<br>Strand-Seq<br>Hi-C<br>PacBio<br>Oxford Nanopore (HG00733)	245<br>113<br>4<br>7<br>17<br>38<br>19	No dedicated SV caller<br>Home made strategy based on haplotype assembly and alignment on reference genome	
Zook et al 2019 [2]	HG002	Illumina HiSeq	300	Spirale Genetics tools, GATK-HC,Freebayes, Fermikits, MetaSV, TNscope, Scalpel, SvABA, Krunch, Cortex,Manta, Seven Graph Bridge Refinement	Optical mapping<br>(Bionano and Nabsys)<br>Assembly with SVanalyzer
		10X Genomics<br>Complete Genomics<br>PacBio	86<br>100<br>44	LongRanger<br>CGATools<br>PbSv<br>Assembly and alignment (Assemblytics)<br>Hybrid : HySA, BreakScan	

2 Characterization of insertion callsets

2.1 Annotation of insertions

Table 2 Annotation of the insertion callset of individual NA19240 according to the minimal sequence coverage threshold. Bracketed values correspond to the category percentage among the annotated insertions.

% Coverage	100	95	80	60	40
**New sequence**	677 (10%)	686 (6%)	869 (6%)	1,223 (8%)	1,639 (11%)
**Mobile element**	605 (9%)	2,047 (17%)	2,473 (18%)	2,828 (19%)	3,321 (22%)
**Tandem repeat**	4,399 (65%)	7,552 (62%)	8,735 (63%)	9,102 (62%)	9,235 (61%)
**Tandem duplication**	444 (7%)	953 (8%)	1,000 (7%)	1,081 (7%)	1,082 (7%)
**Dispersed duplication**	486 (7%)	816 (7%)	774 (6%)	767 (5%)	713 (5%)
**Unassigned**	8,890	3,456	1,843	1,046	473
**% annotated**	43.4	78.0	88.3	93.3	97.0

2.2 Distributions of insertion variant features across several callsets

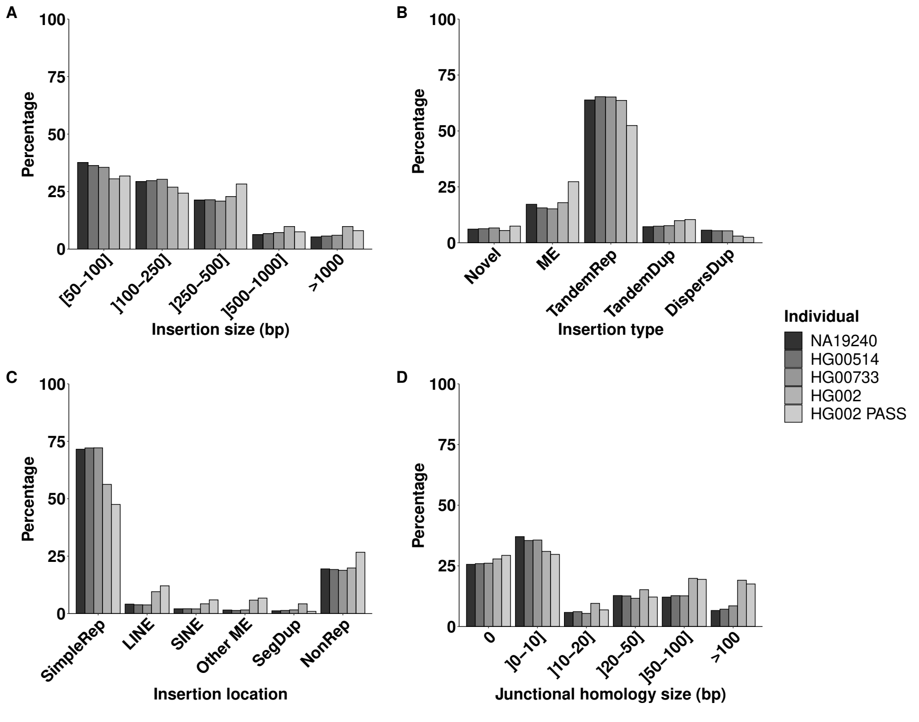

Figure 1 Distributions of insertion variant features across several call sets. Distributions of (A) insertion size, (B) insertion type, (C) repeated context of insertion and (D) homology size at the breakpoint for five insertion variant callsets. NA19240, HG00514, HG00733 refer to the three individual callsets of the Chaisson et al study. HG002 and HG002 PASS refer to the callset of the GiaB study, taking into account, respectively, the full callset or only the variants with PASS in the Filter field. Abbreviations: SimpleRep for simple repeat, ME for mobile element, TandemRep for tandem repeat, TandemDup for tandem duplication, DispersDup for dispersed duplication.

2.3 Proportions of SR-based insertion discoveries according to insertion features

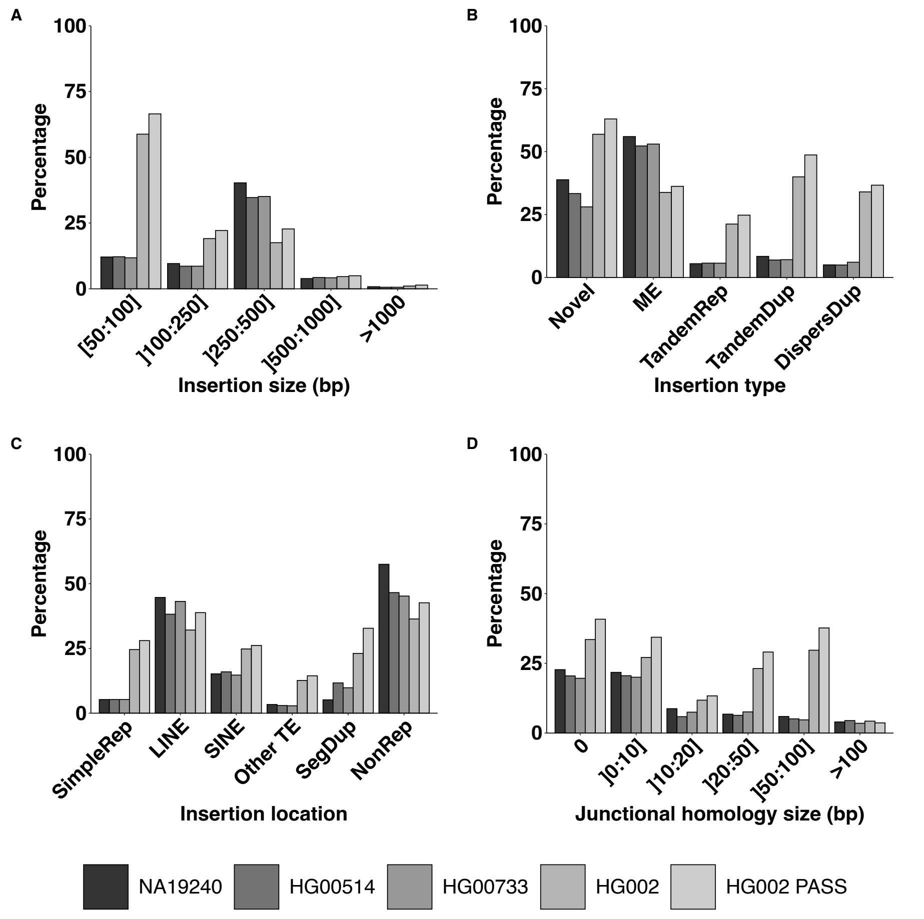

Figure 2 Proportions of SR-based insertion discoveries according to insertion features for the four insertion callsets. Proportions of SR-based insertion discoveries (Short read technology) according to (A) insertion size, (B) insertion type, (C) insertion location and (D) homology size at the breakpoint, in the three individual callsets from the Chaisson et al study (NA19240, HG00514, HG00733) and the HG002 callset from the GiaB study. HG002 PASS refers to the callset of the GiaB study taking into account only the variants with PASS in the Filter field.

3 Additional simulation results

3.1 Recall of SV callers without any quality filter

Table 3 Insertion site recall of several SV callers without any quality filter applied. For each SV caller, all predicted calls output in the final vcf file were taken into account regardless of their value in the FILTER field. Only the insertion site location is taken into account to compute the recall. Each line corresponds to a distinct simulation scenario. Cells of the table are colored according to the variation of the recall value of the given tool with respect to the recall obtained with the baseline simulation (first line, colored in blue): cells in red show a loss of recall >10%, cells in grey show no difference compared to baseline recall at +/- 10%.

		Insertion site only recall - no quality filter (%)			
		**GRIDSS**	**Manta**	**SvABA**	**MindTheGap**
**Baseline simulation**: 250 bp novel sequences in exons		100	100	100	100
**Scenario 1 Insertion size**	50 bp	100	100	100	100
	500 bp	100	86	6	99
	1,000 bp	100	88	1	98
**Scenario 2 Insertion type**	Dispersed duplication	100	49	100	96
	Tandem duplication	100	100	100	0
	Mobile element	100	50	100	58
	Tandem repeat (6 bp pattern)	100	92	22	0
	Tandem repeat (25 bp pattern)	100	66	100	2
**Scenario 3 Junctional homology**	10 bp	100	100	98	0
	20 bp	100	100	89	0
	50 bp	100	51	65	0
	100 bp	100	12	100	0
	150 bp	100	0	100	0
**Scenario 4 Genomic location**	Non repeat	100	100	98	83
	Simple repeat (<300 bp)	100	100	100	73
	Simple repeat (>300 bp)	99	94	100	58
	SINE	100	100	100	53
	LINE	100	100	100	90
	Distance between insertion <150 bp	100	85	77	77
	Real locations	96	81	90	38
**Scenario 5: real insertions at real locations**		65	37	70	6

3.2 False positive amounts

Table 4 Amounts of false positives called by the tested SV callers according to different simulation scenarios. For each scenario involving several simulated datasets, the values indicate the minimal and maximal number of false positive predictions obtained over these datasets. Cells of the table are colored according to the variation of the FP amount of the given tool with respect to the amount obtained with the baseline simulation (first line, colored in blue): cells in red show a substantial increase of FP amount, cells in grey show small difference or a decrease of FP amount compared to the baseline simulation.

	Amount of False positive calls							
	GRIDSS		Manta		SvABA		MindTheGap	
	PASS	All	PASS	All	PASS	All	PASS	All
**Baseline simulation**	0	151	2	2	6	84	19	19
**Scenario 1: Insertion size**	0	131 - 138	0 - 3	0 - 3	0 - 6	82 - 96	16 - 19	16 - 19
**Scenario 2: Insertion type**	3 - 400	233 - 591	0 - 18	0 - 201	4 - 451	92 - 1,157	17 - 19	17 - 19
**Scenario 3: Junctional homology**	2 - 9	128 - 163	0 - 4	0- 4	5 - 202	70 - 342	2 - 18	2 - 18
**Scenario 4: Genomic location**	0 - 4	143 - 166	0 - 5	0 - 5	4 - 13	74 - 643	16 - 19	16 - 19
**Scenario 5: Real insertions**	382	2,052	101	148	523	9,314	19	19

3.3 Insertion recall of short read vs long read SV callers

Table 5 Insertion site and sequence resolved recalls of a short-read insertion caller, GRIDSS, and a long-read insertion caller, Sniffles, according to different simulation scenarios. Cells of the table are colored according to the variation of the recall value of the given tool with respect to the recall obtained with the baseline simulation (first line, colored in blue): cells in red show a loss of recall >10%, cells in grey show no difference compared to baseline recall at +/- 10%. Sequence-resolved recalls were computed with a sequence identity threshold of 90 %, except for the numbers in brackets for which the threshold was lowered to 80 %.

		**Insertion site only recall (%)**		**Sequence resolved recall (%)**	
		**short reads GRIDSS**	**long reads Sniffles**	**short reads GRIDSS**	**long reads Sniffles**
**Baseline simulation**: 250 bp novel seq. in exons		83	100	81	27 (100)
**Scenario 1 Insertion size**	50 bp	56	100	56	33 (100)
	500 bp	100	100	0	19 (100)
	1,000 bp	100	100	0	15 (100)
**Scenario 2 Insertion type**	Dispersed duplication	100	100	0	20 (100)
	Tandem duplication	100	15	0	0 (8)
	Mobile element	100	100	0	23 (100)
	Tandem repeat (6 bp pattern)	100	100	0	14 (100)
	Tandem repeat (25 bp pattern)	99	95	0	10 (95)
**Scenario 3 Junctional homology**	10 bp	100	100	99	9 (100)
	20 bp	100	91	100	5 (90)
	50 bp	77	47	6	2 (42)
	100 bp	100	24	0	0 (11)
	150 bp	100	11	0	0 (6)
**Scenario 4 Genomic location**	Non repeat	83	100	80	22 (100)
	Simple repeat (<300 bp)	82	100	77	25 (100)
	Simple repeat (>300 bp)	87	100	77	19 (100)
	SINE	90	100	77	21 (100)
	LINE	80	100	76	25 (100)
	Clustered insertions (<150 bp)	85	54	75	8 (45)
**Scenario 5 Real insertions**	Novel sequences at real locations	84	58	64	15 (46)
	Real insertions in exonic regions	84	98	11	5 (21)
	Real insertions at real locations	39	58	6	7 (49)

Table 6 Comparison of Sniffles recall values obtained with different validation methods. Recalls in the first two columns were obtained with the methodology described in the main manuscript (ie. +/- 10 bp for insertion site recalls and at least 90 % identity for sequence-resolved recalls). Recalls in the third column named SVanalyzer were obtained with the validation tool developped by GiaB, SVanalyzer/SVbenchmark with option -maxdist set to 10 bp and -minsize set to 50. Cells of the table are colored according to the variation of the recall value of the given tool with respect to the recall obtained with the baseline simulation (first line, colored in blue): cells in red show a loss of recall >10%, cells in grey show no difference compared to baseline recall at +/- 10%. Sequence-resolved recalls were computed with a sequence identity threshold of 90 %, except for the numbers in brackets for which the threshold was lowered to 80 %.

		Method from the study		SVanalyzer
		**Site only (%)**	**Sequence resolved (%)**	**Recall**
**Baseline simulation**: 250 bp novel seq. in exons		100	27 (100)	99.5
**Scenario 1 Insertion size**	50 bp	100	33 (100)	100
	500 bp	100	19 (100)	99.5
	1,000 bp	100	15 (100)	99.5
**Scenario 2 Insertion type**	Dispersed duplication	100	20 (100)	99.5
	Tandem duplication	15	0 (8)	15
	Mobile element	100	23 (100)	99.5
	Tandem repeat (6 bp pattern)	100	14 (100)	99.5
	Tandem repeat (25 bp pattern)	95	10 (95)	99.5
**Scenario 3 Junctional homology**	10 bp	100	9 (100)	99.5
	20 bp	91	5 (90)	90
	50 bp	47	2 (42)	47
	100 bp	24	0 (11)	24
	150 bp	11	0 (6)	11
**Scenario 4 Genomic location**	Non repeat	100	22 (100)	100
	Simple repeat (<300 bp)	100	25 (100)	99.5
	Simple repeat (>300 bp)	100	19 (100)	99.5
	SINE	100	21 (100)	99.5
	LINE	100	25 (100)	99.5
	Clustered insertions (<150 bp)	54	8 (45)	54
**Scenario 5 Real insertions**	Novel sequences at real locations	80	15 (78)	81
	Real insertions in exonic regions	98	5 (21)	98
	Real insertions at real locations	58	7 (49)	57

Author details
[1]Univ Rennes, CNRS, Inria, IRISA, UMR 6074, F-35000 Rennes, France. [2]Unité de Génétique Clinique,Pôle Couple Enfant, CHU de Grenoble Site Nord-Hôpital Couple-Enfant, 38043 Grenoble, France.

References

1. Chaisson, M.J.P., Sanders, A.D., ..., Marschall, T., Korbel, J., Eichler, E.E., Lee, C.: Multi-platform discovery of haplotype-resolved structural variation in human genomes. Nature Communications **10**, 1784 (2019). doi:10.1038/s41467-018-08148-z
2. Zook, J.M., Hansen, N.F., Olson, N.D., Chapman, L., Mullikin, J.C., Xiao, C., Sherry, S., Koren, S., Phillippy, A.M., Boutros, P.C., *et al.*: A robust benchmark for detection of germline large deletions and insertions. Nature Biotechnology (2020). doi:10.1038/s41587-020-0538-8
